# International Dental Congress

**Published:** 1902-12

**Authors:** 


### INTERNATIONAL DENTAL CONGRESS.

The committee having this matter in charge has been actively at work endeavoring to bring order out of a chaotic condition of things.

The committee appointed by the Féderation Dentaire Internationale to take charge of its interests in this country in connection with the congress is still persistent in claiming precedence in the matter, allusion to which was made in our last issue.

It seems, from correspondence, that at the Third International Dental Congress, held in Paris, 1900, the Executive Council of the Féderation Dentaire Internationale was by resolution "intrusted with the organization and preparation of the next congress."

The International Dental Congress held at Chicago, 1893, was called by the united action of the American Dental Association and the Southern Dental Association. When it closed its labors we are not aware that it delegated authority to any body to call another congress.

When the call was issued for the International Dental Congress to be held in Paris no allusion was made to any previous authority to issue said call, nor was there any necessity for it. These International Dental Congresses have heretofore been held during the period of certain great expositions, and, therefore, any definite time for the holding is entirely out of the question.

In view of these uncertain periods, it seems proper that the country in which the exposition is held should have the entire management of the congress. This has, heretofore, been the case, and this departure from established usage is not at all in accordance with international comity.

A congress has, without doubt, the right to delegate the power to any body to call together any subsequent congress, but it is equally the privilege of any nationality to decline or accept such authority. The Fédération Dentaire Internationale is a body of dental educators from various sections of the civilized world, While men of broad intelligence, it does not seem that this is exactly the organization to manage an international congress.

The proposed congress of 1904 was ordered by the National Dental Association at its meeting at Niagara Falls, 1902, and in its invitation to the Fédération it expected and desired that it should work conjointly with it to make the congress a success, but the members were not aware of the aforesaid resolution empowering the Fédération to call and manage the congress. Dr. Godon, president of the Fédération Dentaire Internationale, has apparently the correct idea, for he says, "It seems to us that the committee of nine should hold a meeting, appoint officers (President, Vice-Presidents, and Secretaries), and the committee thus formed should put itself in relation with the several societies that addressed the invitations, so as to *collaborate* to the good organization of the congress and will place itself at the disposition of the organizers." If this is carried out by the committee all further trouble will be avoided, but its present attitude, if persisted in, will not only injure the prosperity of the congress, but will create antagonistic feelings that years may fail to remove. The true solution of this whole matter is to avoid all interference by other nationalities with the management of a congress, no matter where held, leaving it entirely with those most familiar with local conditions and national peculiarities.

